# Frequency of malnutrition in adult patients with stage V chronic kidney disease on hemodialysis

**DOI:** 10.17843/rpmesp.2024.414.13638

**Published:** 2024-10-21

**Authors:** Luis Ángel Rodríguez-Chávez, Solessi Ramírez-Pachamango, Cristhian Renzho Elsayed Rodríguez-Mendoza

**Affiliations:** 1 Antenor Orrego Private University, Trujillo, Peru. Antenor Orrego Private University Antenor Orrego Private University Trujillo Peru; 2 Internal Medicine Research Center of the High Complexity Hospital of La Libertad “Virgen de la Puerta”, Trujillo, Peru. Internal Medicine Research Center of the High Complexity Hospital of La Libertad “Virgen de la Puerta” Trujillo Peru

**Keywords:** Malnutrition, Chronic Kidney Failure, Hemodialysis

## Abstract

This was a cross-sectional observational study that aimed to determine the frequency of malnutrition in adult patients with stage V chronic kidney disease on hemodialysis. The sample consisted of 105 adult patients diagnosed stage V chronic kidney disease who received hemodialysis at the “Virgen de la Puerta” High Complexity Hospital in Trujillo, Peru. We applied the Malnutrition-Inflammation Score (MIS), 97 (92.4%) patients presented malnutrition, of which 20% had mild malnutrition, 37.1% had moderate malnutrition and 35.3% had severe malnutrition; only 8 patients (7.6%) presented normal nutritional status. In conclusion, we found a high frequency of malnutrition among patients diagnosed with stage V chronic kidney disease receiving hemodialysis.

## INTRODUCTION

Chronic kidney disease (CKD) is considered a worldwide public health problem, it is a clinical syndrome secondary to abnormalities of renal function and/or structure and is characterized by being slow, progressive and irreversible ^1^^,^^2^^,^^3^. A person is considered to have CKD when he/she maintains a glomerular filtration rate (GFR) of <60 ml/min/1.73 m^2^ or damage of the renal structure evidenced in laboratory markers or images for three months or more. CKD usually progresses to more advanced stages and can even lead to death, therefore early and adequate diagnosis and management is essential ^1^^,^^2^.

CKD has a global prevalence of 11 to 13%, with an estimated prevalence of grade V of 0.1% ^4^. In Peru, CKD is considered one of the main causes of death and its prevalence in some regions is estimated to be approximately 16.8%, while the prevalence of grade V is 0.2% ^4^. According to data provided by the Social Health Insurance (EsSalud) of La Libertad, the number of patients receiving hemodialysis is increasing, with approximately 150 new patients per year. In 2023, 1567 hemodialysis sessions were performed at the “Virgen de la Puerta” high complexity hospital, almost all of which corresponded to CKD, with an approximate cost of US$ 12,000 per patient per year ^5^.

Patients who suffer from the most advanced grades of this disease (IV and V), are those who present the majority of complications due to the decrease in GFR; these complications include arterial hypertension, anemia, bone mineral disorder, neuropathy and malnutrition ^6^. If, in addition, they receive renal replacement therapy, it may cause metabolic and nutritional alterations that involve chronic inflammation and protein catabolism, negatively affecting nutritional status ^7^; in addition, in patients undergoing hemodialysis, although this therapy eliminates most of the wastes in a short time, it can also eliminate important nutrients for the body. In this group of patients, the prevalence of malnutrition ranges from 10 to 70% depending on the diagnostic tool ^8^. Malnutrition is a risk factor that increases patient morbidity and mortality regardless of the pathology with which it is associated (clinical or surgical) ^8^.

The objective of this study was to determine the frequency of malnutrition in patients with CKD grade V on hemodialysis. The importance of this study lies in the fact that it allows us to obtain data on the high prevalence of malnutrition in patients receiving hemodialysis as renal replacement therapy, as well as to identify patients for nutritional intervention as the start of activities of the metabolic-nutritional support unit of our hospital.

**KEY MESSAGES**

Motivation for the study. The frequency of malnutrition in adult patients with grade V chronic kidney disease on hemodialysis at the High Complexity Hospital of La Libertad “Virgen de la Puerta” is unknown.

Main findings. We found high frequency of malnutrition in hemodialysis patients (92.4%), with moderate/severe malnutrition predominating (72.4%).

Implications. This study allows us to understand the situation of malnutrition in hemodialysis patients in order to initiate early nutritional intervention, in addition to providing important data that add to the available evidence.

## THE STUDY

### Study design and population

Analytical cross-sectional study carried out in the hemodialysis unit of the Hospital de Alta Complejidad “Virgen de la Puerta”. This hospital is part of EsSalud, has category oncology III E; that is to say, a third level hospital specialized in oncology, with attention, in addition to other conditions, that require the capacity of third level care. This hospital is a referral center for the health care networks of La Libertad, Cajamarca, Tarapoto and Ancash. It treats patients with multiple conditions, including cardiometabolic and oncologic diseases.

### Selection criteria

The inclusion criteria were patients older than 18 years of age, of both sexes, who attended the hemodialysis program regularly (they complied with the frequency of sessions established by medical indication). Patients who decided to voluntarily withdraw from the study and pregnant patients were excluded.

### Sample size

The sample was of the census type and consisted of the entire population, 105 patients in the regular hemodialysis program ^5^, there were no patients excluded, since all of them agreed to participate in the study after explaining that it was about the actions of the metabolic nutritional support unit of the hospital, there were no pregnant patients.

### Data collection

The interview was conducted with the patient and their family member in the hemodialysis unit of the hospital, where the type of study and its methodology were explained to them, they were also informed that the study was part of the program to initiate the activities of the Metabolic/Nutritional Support Unit (USMEN) in the hemodialysis unit. After agreeing to participate in the study, the patient and their family member signed the informed consent form. Then, they were asked the questions of the data collection form and the Malnutrition-Inflammation Scale (MIS). Finally, the medical records were reviewed to complete the missing data from the MIS ^7^.

### Variables

Malnutrition was measured with the Malnutrition-Inflammation Scale or Malnutrition-Inflammation Score (MIS) which includes 10 parameters: dry weight change at the end of hemodialysis, dietary intake, gastrointestinal symptoms, functional capacity, comorbidity including years of dialysis, decrease in fat deposits or loss of subcutaneous fat, signs of muscle mass loss, body mass index, serum albumin and serum transferrin; divided into four sections: nutritional history, physical examination, body mass index and laboratory parameters. A score of less than 3 points is considered normal, 3 to 5 points is mild malnutrition, 6 to 8 points is moderate malnutrition and a score of more than 8 points represents severe malnutrition. This scale has been validated in patients with chronic renal disease on hemodialysis ^7^.

In addition, variables such as age, sex, origin, polypharmacy (use of more than four medications per day), time of diagnosis of chronic kidney disease, time in the hemodialysis program, number of hospitalizations in the last year were included.

### Statistical Analysis

The information was analyzed with the SPSS V25.0 statistical package. The results are presented in frequencies and percentages in simple and crossed tables for qualitative variables.

### Ethical Aspects

This study was approved by the Bioethics Committee of the Universidad Privada Antenor Orrego (bioethics committee resolution N°0770-2023-UPAO), and by the Ethics Committee of the Office of Training, Research and Teaching of the EsSalud Healthcare Network - La Libertad (P.I N° 119 CIYE-O.C.I Y D-RALL-ESSALUD-2023). In addition, the patient and accompanying family member signed the informed consent form.

## RESULTS

Table 1 shows the sociodemographic characteristics of the patients with CKD grade V on hemodialysis, 52.4% (n=55) were male and 47.6% (n=50) were female. Most of the patients were older than 60 years (57.1%; n=60), had high school education (45.7%; n=48) and married (54.3%; n=57).

**Table 1:** Sociodemographic characteristics of adult patients with grade V chronic kidney disease on hemodialysis grouped according to nutritional status.

Variable		Total	Normal	Mild malnutrition	Moderate malnutrition	Severe malnutrition
		n (%)	n (%)	n (%)	n (%)	n (%)
Age						
	Under 60 years of age	45 (42.9)	5 (62.5)	11 (52.4)	19 (48.7)	10 (27.0)
	60 years of age and older	60 (57.1)	3 (37.5)	10 (47.6)	20 (51.3)	27 (73.0)
Sex						
	Male	55 (52.4)	6 (75.0)	13 (61.9)	21 (53.8)	15 (40.5)
	Female	50 (47.6)	2 (25.0)	8 (38.1)	18 (46.2)	22 (59.5)
Education level						
	No education	2 (1.9)	0 (0.0)	0 (0.0)	0 (0.0)	2 (5.4)
	Primary school	24 (22.9)	1 (12.5)	3 (14.2)	8 (20.5)	12 (32.4)
	Secondary school	48 (45.7)	6 (75.0)	9 (42.9)	18 (46.2)	15 (40.6)
	Higher education	31 (29.5)	1 (12.5)	9 (42.9)	13 (33.3)	8 (21.6)
Marital status						
	Single	17 (16.2)	2 (25.0)	0 (0.0)	9 (23.1)	6 (16.2)
	Married	57 (54.3)	4 (50.0)	14 (66.7)	21 (53.8)	18 (48.7)
	Divorced	6 (5.7)	1 (12.5)	0 (0.0)	2 (5.1)	3 (8.1)
	Cohabitant	13 (12.4)	0 (0.0)	4 (19.0)	6 (15.4)	3 (8.1)
	Widower	12 (11.4)	1 (15.5)	3 (14.3)	1 (2.6)	7 (18.9)
Place of origin						
	Rural	38 (36.2)	2 (25.0)	5 (23.8)	14 (35.9)	17 (45.9)
	Urban	67 (63.8)	6 (75.0)	16 (76.2)	25 (64.1)	20 (54.1)

Table 2 shows that 7.6% (n=8) of patients had normal nutritional status, while 20% (n=21) presented mild malnutrition, 37.1% (n=39) moderate malnutrition and 35.5% (n=37) severe malnutrition. The most frequent comorbidity was arterial hypertension with 93.3% (n=98), type 2 diabetes mellitus with 53.3% (n=56), and polypharmacy with 68.6% (n=72).

**Table 2:** Comorbidities and characteristics of adult patients with grade V chronic kidney disease on hemodialysis grouped according to nutritional status.

Variable		Total	Normal	Mild malnutrition	Moderate malnutrition	Severe malnutrition
		n (%)	n (%)	n (%)	n (%)	n (%)
Diabetes mellitus type 2						
	No	49 (46.7)	6 (12.2)	9 (18.4)	16 (32.7)	18 (36.7)
	Yes	56 (53.3)	2 (3.6)	12 (21.4)	23 (41.0)	19 (33.9)
Arterial hypertension						
	No	7 (6.7)	1 (14.3)	0 (0.0)	4 (57.1)	2 (28.6)
	Yes	98 (93.3)	7 (7.2)	21 (21.4)	35 (35.7)	35 (35.7)
Polypharmacy						
	No	33 (31.4)	3 (9.1)	2 (6.1)	18 (54.5)	10 (30.3)
	Yes	72 (68.6)	5 (6.9)	19 (26.4)	21 (29.2)	27 (37.5)
Time to diagnosis of grade V CKD						
	Less than 1 year	7 (6.7)	1 (14.2)	0 (0.0)	3 (42.9)	3 (42.9)
	From 1 to 4 years	39 (37.1)	5 (12.9)	8 (20.5)	16 (41.0)	10 (25.6)
	More than 4 years	59 (56.2)	2 (3.4)	13 (22.0)	20 (33.9)	24 (40.7)
Time on hemodialysis program						
	Less than 1 year	24 (22.9)	3 (12.5)	4 (16.7)	8 (33.3)	9 (37.5)
	From 1 to 4 years	48 (45.7)	5 (10.4)	9 (18.7)	21 (43.8)	13 (27.1)
	More than 4 years	33 (31.4)	0 (0.0)	8 (24.2)	10 (30.3)	15 (45.5)
Number of hemodialysis sessions per week						
	1	1 (1.0)	0 (0.0)	0 (0.0)	0 (0.0)	1 (100.0)
	2	2 (1.9)	0 (0.0)	0 (0.0)	1 (50.0)	1 (50.0)
	3	102 (97.1)	8 (7.8)	21 (20.6)	38 (37.3)	35 (34.3)
Number of hospitalizations in the last year						
	Less than 3	84 (80.0)	7 (8.3)	18 (21.4)	34 (40.5)	25 (29.8)
	3 or more	21 (20.0)	1 (4.7)	3 (13.4)	5 (23.8)	12 (57.1)

CKD: chronic kidney disease

Regarding the characteristics of CKD and its treatment, 56.2% (n=59) of patients had been diagnosed with CKD for more than four years, 31.4% (n=33) of patients had been receiving hemodialysis for more than four years and 97.1% (n=102) of patients attended three hemodialysis sessions per week, 80% (n=84) of patients had less than three hospital admissions in the last year.

## DISCUSSION

In this study of patients with CKD grade V on hemodialysis (n=105), we found that 52.4% were men and 57.1% were over 60 years of age. Some degree of malnutrition was found in 92.4% of the patients: mild (20%), moderate (37.1%) or severe (35.5%). The most common comorbidities were arterial hypertension (93.3%) and diabetes mellitus type 2 (53.3%). Of the patients, 56.2% had been diagnosed with CKD for more than four years, 97.1% attended three hemodialysis sessions per week, and 80% had less than three hospitalizations in the last year.

When it comes to the nutritional status of the patients, we found that only 7.6% had normal nutritional status, while 92.4% showed some degree of malnutrition. This coincides with that reported by Vásquez *et al*. who, in a study carried out in Chiclayo (Peru), found that 4% of their patients had normal nutritional status, while 96% showed some degree of malnutrition ^10^. These similar figures could be attributed to the use of the same scale and, in particular, to the use of the same cut-off point (score greater than or equal to 3) to determine the presence of malnutrition in this group of patients. However, the frequency of malnutrition varies when different measurement scales are used. For example, Oliveira *et al*. and Rodriguez *et al*. who used the Subjective Global Rating (GSR) scale reported a frequency of malnutrition of 19.5% and 34.0%, respectively ^11^^,^^12^.

The percentage of malnutrition was slightly higher in patients older than 63 years, which is consistent with that reported by Gómez *et al*. and Munive *et al*. who also used the MIS scale in studies with similar sample sizes. Other authors have found that age is significantly associated with malnutrition, with patients older than 60 years having higher prevalence of malnutrition ^11^^-^^15^.

Regarding the relationship between sex and malnutrition, we found that 46.7% of men had malnutrition, compared to 45.7% of women. Elliot *et al*. reported that sex did not have a significant association, therefore it is not a factor affecting nutritional status ^16^. Similarly, Oliveira *et al*., Omari *et al*., and Freitas *et al*. reported that there was no significant association between sex and malnutrition ^11^^,^^14^^,^^18^. In addition, patients consuming four or more medications had higher frequency of malnutrition than those consuming less than four medications. This finding coincides with the study by Omari *et al*. which reported that the greater the number of medications consumed, the greater the susceptibility to malnutrition ^14^.

In addition, malnutrition in CKD patients on hemodialysis could be explained by other factors not indicated in this study, such as loss of appetite, dietary restriction, chronic inflammation, oxidative stress, and loss of protein and calories during the dialytic procedure ^19^^,^^20^.

One of the main limitations of this study is the fact that it was carried out in a single hemodialysis center of the La Libertad health care network, with a small sample, so the number of patients with normal nutritional status was very low compared to the number of patients with malnutrition, which prevents an adequate comparison. In addition, the study population is insured, which does not necessarily correspond to a homogeneous sample of the entire population with CKD on hemodialysis (insured and uninsured).

In conclusion, it is evident that, in our setting, malnutrition is a highly frequent health problem among CKD grade V patients receiving hemodialysis, with moderate to severe malnutrition predominating. This finding is important since malnutrition is a predictor of morbidity and mortality and various complications. In addition, it is related to poor prognosis, including worse quality of life, refractory anemia, and significantly high rates of hospitalization and mortality.
