# Supplementary material for: Frequency of malnutrition in adult patients with stage V chronic kidney disease on hemodialysis
Source: Rev Peru Med Exp Salud Publica. 2024 Oct 21;41(4):399–404. doi: 10.17843/rpmesp.2024.414.13638 (PMC11797589; doi:10.17843/rpmesp.2024.414.13638)
Supplement: Translation

# Translation: Frecuencia de desnutrición en pacientes adultos con enfermedad renal crónica grado V en hemodiálisis

**Authors:** Luis Ángel Rodríguez-Chávez, Solessi Ramírez-Pachamango, Cristhian Renzho Elsayed Rodríguez-Mendoza

**Affiliations:** 1 Universidad Privada Antenor Orrego, Trujillo, Perú.; 2 Centro de investigación en medicina interna del Hospital de Alta Complejidad de La Libertad «Virgen de la Puerta», Trujillo, Perú.

**Keywords:** Desnutrición, Enfermedad Renal Crónica, Hemodiálisis

## Abstract

El objetivo fue determinar la frecuencia de desnutrición en pacientes adultos con enfermedad renal crónica grado V en hemodiálisis, para ello se realizó un estudio observacional transversal. La muestra estuvo constituida por 105 pacientes adultos con diagnóstico de enfermedad renal crónica grado V que recibieron hemodiálisis en el Hospital de Alta Complejidad «Virgen de la Puerta» de Trujillo, Perú. Se aplicó la Escala Malnutrition-Inflamation Score (MIS), 97 (92,4%) pacientes presentaron desnutrición, dividido en 20% con desnutrición leve, 37,1% con desnutrición moderada y 35,3% con desnutrición grave; solo 8 pacientes (7,6%) presentaron estado nutricional normal. En conclusión, existe una alta frecuencia de desnutrición entre los pacientes con diagnóstico de enfermedad renal crónica grado V que reciben hemodiálisis.

## INTRODUCCION

La enfermedad renal crónica (ERC) se considera un problema mundial de salud pública, consiste en un síndrome clínico secundario a anomalías de la función y/o la estructura renal y tiene como característica una evolución lenta, progresiva e irreversible ^(^^1^^,^^2^^,^^3^. Se considera que una persona tiene ERC cuando mantiene durante tres meses o más, una tasa de filtrado glomerular (TFG) <60 ml/min/1,73 m^2^ o daño de la estructura renal evidenciado en marcadores de laboratorio o imágenes. Esta suele evolucionar hacia etapas más avanzadas pudiendo llegar incluso a la muerte, por lo que es fundamental el diagnóstico y manejo temprano y adecuado ^1^^,^^2^.

La ERC tiene una prevalencia global del 11 al 13%, siendo la prevalencia estimada del grado V del 0,1% ^4^. En el Perú, la ERC es considerada una de las principales causas de muerte y se estima que su prevalencia en algunas de las regiones es aproximadamente del 16,8%, mientras que, la prevalencia en grado V es de 0,2% ^4^. Según datos brindados por el Seguro Social en Salud (EsSalud) de La Libertad, el número de pacientes que reciben hemodiálisis va en aumento, con aproximadamente 150 pacientes nuevos por año. En el 2023, en el hospital de alta complejidad «Virgen de la Puerta» se realizaron 1567 sesiones de hemodiálisis, las que, casi en su totalidad, correspondieron a ERC, con un gasto aproximado de 12 mil dólares por paciente al año ^5^.

Los pacientes que padecen los últimos grados de esta enfermedad (IV y V), son los que presentan la mayoría de complicaciones debido a la disminución de la TFG; estas complicaciones incluyen la hipertensión arterial, anemia, trastorno mineral óseo, neuropatía y desnutrición ^6^. Si, además, reciben terapia de reemplazo renal, se generan alteraciones metabólicas y nutricionales que implican inflamación crónica y catabolismo de proteínas, afectando de manera negativa el estado nutricional ^7^; además se suma que, en los pacientes sometidos a hemodiálisis, si bien dicha terapia elimina la mayor parte de los desechos en poco tiempo, también puede eliminar nutrientes importantes para el organismo. En este grupo de pacientes, la prevalencia de desnutrición oscila entre el 10 al 70% según la herramienta de diagnóstico ^8^. La desnutrición es un factor de riesgo que aumenta la morbimortalidad de los pacientes independientemente de la patología a la que este asociada (sea clínica o quirúrgica) ^(^^9^.

El objetivo del presente estudio fue determinar la frecuencia de la desnutrición en los pacientes con ERC grado V en hemodiálisis; la importancia del presente estudio radica en que permite obtener datos de la prevalencia alta de desnutrición en los pacientes que reciben hemodiálisis como terapia de reemplazo renal, así mismo permite identificar a los pacientes para realizar la intervención nutricional como inicio de actividades de la unidad de soporte metabólico nutricional de nuestro hospital.

**MENSAJE CLAVE**

Motivación para realizar el estudio. Se desconoce la frecuencia de la desnutrición en pacientes adultos con enfermedad renal crónica grado V en hemodiálisis en el Hospital de Alta Complejidad De La Libertad «Virgen de la Puerta».

Principales hallazgos. Existe una alta frecuencia de desnutrición en los pacientes en hemodiálisis (92,4%), predominando la desnutrición moderada/severa (72,4%).

Implicancias. Este estudio permite conocer la situación de la desnutrición en los pacientes en hemodiálisis con la finalidad de iniciar la intervención nutricional temprana, además se brindan datos importantes que se suman a la evidencia disponible.

## EL ESTUDIO

### Diseño y población del estudio

Estudio transversal analítico realizado en la unidad de hemodiálisis del Hospital de Alta Complejidad «Virgen de la Puerta». Este hospital pertenece a EsSalud, tiene la categoría III E oncológico; es decir un hospital del tercer nivel de atención especializado en oncología, con atenciones, además de otras patologías, que requieran de capacidad resolutiva del tercer nivel de atención. Este hospital es centro de referencia de las redes asistenciales de La Libertad, Cajamarca, Tarapoto y Ancash. Atiende pacientes con pluripatologías entre las que destacan las cardiometabólico y oncológicas.

### Criterios de selección

Los criterios de inclusión fueron a pacientes con edad mayor a 18 años, de ambos sexos, que asisten al programa de hemodiálisis regularmente (cumplen la frecuencia de sesiones establecidas por indicación médica). Se excluyeron a pacientes que decidieron retirarse voluntariamente del estudio y a pacientes gestantes**.**

### Tamaño de muestra

La muestra fue de tipo censal y estuvo conformada por la totalidad de la población, 105 pacientes de programa regular de hemodiálisis ^5^, no hubo pacientes excluidos, debido a que todos aceptaron participar en el estudio luego de explicarles que se trataba de las acciones de la unidad de soporte metabólico nutricional del hospital, no hubo pacientes gestantes.

### Recolección de datos

La entrevista se realizó al paciente en compañía de su familiar en la unidad de hemodiálisis del hospital, donde se les explico el tipo de estudio y su metodología, y se les informó que el estudio forma parte del programa de inicio de las actividades de la Unidad de Soporte Metabólico/Nutricional (USMEN) en la unidad de hemodiálisis. El paciente y su familiar luego de aceptar participar en el estudio firmaron el consentimiento informado. Luego, se les realizó las preguntas de la ficha de recolección de datos y de la Escala de Malnutrición-Inflamación (MIS). Finalmente, se revisó las historias clínicas para completar los datos faltantes de la MIS ^7^.

### Variables

La desnutrición se midió con la Escala de Malnutrición-Inflamación o Malnutrition-Inflamation Score (MIS) que incluye 10 parámetros: cambio de peso seco al final hemodiálisis, ingesta alimentaria, síntomas gastrointestinales, capacidad funcional, comorbilidad incluido los años de diálisis, disminución de depósitos de grasa o pérdida de grasa subcutánea, signos de pérdida de masa muscular, índice de masa corporal, albúmina sérica y transferrina sérica; dividido en cuatro apartados: historia nutricional, examen físico, índice de masa corporal y parámetros laboratoriales. Un puntaje menor a 3 es considerado normal, de 3 a 5 puntos es desnutrición leve, de 6 a 8 puntos es desnutrición moderada y un puntaje mayor a 8 es desnutrición grave. Esta escala esta validada en pacientes con enfermedad renal crónica en hemodiálisis ^7^.

Adicionalmente, se incluyeron variables como la edad, sexo, procedencia, polifarmacia (uso de más de cuatro medicamentos al día), tiempo de diagnóstico de enfermedad renal crónica, tiempo en el programa de hemodiálisis, número de hospitalizaciones en el último año.

### Análisis Estadístico

La información fue analizada con el paquete estadístico SPSS V25.0. Los resultados se presentan en frecuencias y porcentajes en tablas simples y cruzadas para las variables cualitativas.

### Aspectos éticos

El presente estudio fue aprobado por el Comité de Bioética de la Universidad Privada Antenor Orrego (resolución comité bioética N°0770-2023-UPAO), y por el Comité de Ética de la Oficina de Capacitación, Investigación y Docencia de la Red Asistencial de EsSalud - La Libertad (P.I N° 119 CIYE-O.C.I Y D-RALL-ESSALUD-2023). Además, el paciente y familiar acompañante firmaron el consentimiento informado.

## RESULTADOS

La tabla 1 muestra las características sociodemográficas de los pacientes con ERC grado V en hemodiálisis, se observa que el 52,4% (n=55) fueron del sexo masculino y el 47,6% (n=50) de sexo femenino. La mayoría de los pacientes eran mayores de 60 años con un 57,1% (n=60), tenían el grado de instrucción secundaria con 45,7% (n=48) y estado civil casado con 54,3% (n=57).

**Tabla 1:** Características sociodemográficas de los pacientes adultos con enfermedad renal crónica grado V en hemodiálisis agrupados según el estado de nutrición.

Variable		Total	Normal	Desnutrición leve	Desnutrición moderada	Desnutrición grave
		n (%)	n (%)	n (%)	n (%)	n (%)
Edad						
	Menor de 60 años	45 (42,9)	5 (62,5)	11 (52,4)	19 (48,7)	10 (27,0)
	Mayor e igual de 60 años	60 (57,1)	3 (37,5)	10 (47,6)	20 (51,3)	27 (73,0)
Sexo						
	Masculino	55 (52,4)	6 (75,0)	13 (61,9)	21 (53,8)	15 (40,5)
	Femenino	50 (47,6)	2 (25,0)	8 (38,1)	18 (46,2)	22 (59,5)
Grado de instrucción						
	Sin estudios	2 (1,9)	0 (0,0)	0 (0,0)	0 (0,0)	2 (5,4)
	Primaria	24 (22,9)	1 (12,5)	3 (14,2)	8 (20,5)	12 (32,4)
	Secundaria	48 (45,7)	6 (75,0)	9 (42,9)	18 (46,2)	15 (40,6)
	Superior	31 (29,5)	1 (12,5)	9 (42,9)	13 (33,3)	8 (21,6)
Estado civil						
	Soltero	17 (16,2)	2 (25,0)	0 (0,0)	9 (23,1)	6 (16,2)
	Casado	57 (54,3)	4 (50,0)	14 (66,7)	21 (53,8)	18 (48,7)
	Divorciado	6 (5,7)	1 (12,5)	0 (0,0)	2 (5,1)	3 (8,1)
	Conviviente	13 (12,4)	0 (0,0)	4 (19,0)	6 (15,4)	3 (8,1)
	Viudo	12 (11,4)	1 (15,5)	3 (14,3)	1 (2,6)	7 (18,9)
Procedencia						
	Rural	38 (36,2)	2 (25,0)	5 (23,8)	14 (35,9)	17 (45,9)
	Urbano	67 (63,8)	6 (75,0)	16 (76,2)	25 (64,1)	20 (54,1)

La tabla 2 se muestra que el 7,6% (n=8) de pacientes presentaron un estado nutricional normal, mientras que el 20% (n=21) presentó desnutrición leve, el 37,1% (n=39) desnutrición moderada y el 35,5% (n=37) desnutrición grave. Sobre las comorbilidades, la más frecuente fue la hipertensión arterial con el 93,3% (n=98), diabetes mellitus tipo 2 con 53,3% (n=56), y polifarmacia con 68,6% (n=72).

**Tabla 2:** Comorbilidades y características de los pacientes adultos con enfermedad renal crónica grado V en hemodiálisis agrupados según el estado de nutrición.

Variable		Total	Normal	Desnutrición leve	Desnutrición moderada	Desnutrición grave
		n (%)	n (%)	n (%)	n (%)	n (%)
Diabetes mellitus tipo 2						
	No	49 (46,7)	6 (12,2)	9 (18,4)	16 (32,7)	18 (36,7)
	Si	56 (53,3)	2 (3,6)	12 (21,4)	23 (41,0)	19 (33,9)
Hipertensión arterial						
	No	7 (6,7)	1 (14,3)	0 (0,0)	4 (57,1)	2 (28,6)
	Si	98 (93,3)	7 (7,2)	21 (21,4)	35 (35,7)	35 (35,7)
Polifarmacia						
	No	33 (31,4)	3 (9,1)	2 (6,1)	18 (54,5)	10 (30,3)
	Si	72 (68,6)	5 (6,9)	19 (26,4)	21 (29,2)	27 (37,5)
Tiempo de diagnóstico de ERC grado V						
	Menor a 1 año	7 (6,7)	1 (14,2)	0 (0,0)	3 (42,9)	3 (42,9)
	De 1 a 4 años	39 (37,1)	5 (12,9)	8 (20,5)	16 (41,0)	10 (25,6)
	Mayor a 4 años	59 (56,2)	2 (3,4)	13 (22,0)	20 (33,9)	24 (40,7)
Tiempo en programa de hemodiálisis						
	Menor a 1 año	24 (22,9)	3 (12,5)	4 (16,7)	8 (33,3)	9 (37,5)
	De 1 a 4 años	48 (45,7)	5 (10,4)	9 (18,7)	21 (43,8)	13 (27,1)
	Mayor a 4 años	33 (31,4)	0 (0,0)	8 (24,2)	0 (30,3)	15 (45,5)
Número de sesiones de hemodiálisis por semana						
	1	1 (1,0)	0 (0,0)	0 (0,0)	0 (0,0)	1 (100,0)
	2	2 (1,9)	0 (0,0)	0 (0,0)	1 (50,0)	1 (50,0)
	3	102 (97,1)	8 (7,8)	21 (20,6)	38 (37,3)	35 (34,3)
Número de hospitalizaciones en el último año						
	Menos de 3	84 (80,0)	7 (8,3)	18 (21,4)	34 (40,5)	25 (29,8)
	3 o más	21 (20,0)	1 (4,7)	3 (13,4)	5 (23,8)	12 (57,1)

ERC: enfermedad renal crónica

En cuanto a las características de la ERC y su tratamiento, el 56,2% (n=59) de pacientes tenían más cuatro años con el diagnóstico de ERC, el 31,4% (n=33) de los pacientes llevaban más de cuatro años recibiendo hemodiálisis y el 97,1% (n=102) de pacientes asistían a tres sesiones de hemodiálisis por semana, el 80% (n=84) de pacientes tuvieron menos de tres ingresos a hospitalización en el último año.

## DISCUSIÓN

En este estudio de pacientes con ERC grado V en hemodiálisis (n=105), el 52,4% fueron hombres y el 57,1% mayores de 60 años. El 92,4% presentó algún grado de desnutrición: leve (20%), moderada (37,1%) o grave (35,5%). Las comorbilidades más comunes fueron hipertensión arterial (93,3%) y diabetes mellitus tipo 2 (53,3%). El 56,2% de los pacientes tenían más de cuatro años con diagnóstico de ERC, el 97,1% asistía a tres sesiones de hemodiálisis por semana, y el 80% tuvo menos de tres hospitalizaciones en el último año.

 En relación con el estado nutricional de los pacientes, se observó que solo el 7,6% presentaba un estado nutricional normal, mientras que el 92,4% mostraba algún grado de desnutrición. Esto coincide con lo reportado por Vásquez *et al*., quienes, en un estudio realizado en Chiclayo (Perú), encontraron que el 4% de sus pacientes tenía un estado nutricional normal, mientras que el 96% presentaba algún grado de desnutrición ^10^. Estas cifras similares podrían atribuirse al uso de la misma escala y, en particular, al empleo del mismo punto de corte (puntaje mayor o igual a 3) para determinar la presencia de desnutrición en este grupo de pacientes. Sin embargo, la frecuencia de desnutrición varía cuando se emplean diferentes escalas de medición. Por ejemplo, Oliveira *et al*. y Rodríguez *et al*., quienes utilizaron la escala de Valoración Global Subjetiva (VGS), reportaron una frecuencia de desnutrición del 19,5% y 34,0%, respectivamente ^11^^,^^12^.

El porcentaje de desnutrición fue ligeramente superior en pacientes mayores de 63 años, lo que concuerda con lo reportado por Gómez *et al*. y Munive *et al*., quienes también emplearon la escala MIS en estudios con tamaños de muestras similares. Otros autores han encontrado que la edad se asocia significativamente con la desnutrición, siendo los pacientes mayores de 60 años quienes presentan una mayor prevalencia de desnutrición ^11^^-^^15^.

En cuanto a la relación entre sexo y desnutrición, se observó que el 46,7% de los hombres tenían desnutrición, en comparación con el 45,7% de las mujeres. Elliot *et al*. reportaron que el sexo no mostró una asociación significativa, por lo que no se considera un factor que afecta el estado nutricional ^16^. De manera similar, Oliveira *et al*., Omari *et al*., y Freitas *et al*. informaron que no hubo una asociación significativa entre el sexo y la desnutrición ^11^^,^^17^^,^^18^. Además, se observó que los pacientes que consumían cuatro o más medicamentos presentaban una frecuencia de desnutrición mayor que aquellos que consumían menos de cuatro medicamentos. Este hallazgo coincide con el estudio de Omari *et al*., en el cual se reporta que, a mayor número de medicamentos consumidos, mayor es la susceptibilidad a la desnutrición ^14^.

Además, la desnutrición en pacientes con ERC en hemodiálisis podría estar explicada por otros factores no indicados en este estudio, como la pérdida de apetito, la restricción dietética, la inflamación crónica, el estrés oxidativo y la pérdida de proteínas y calorías durante el procedimiento dialítico ^19^^,^^20^.

Como limitaciones se debe reconocer que este estudio se realizó en un solo centro de hemodiálisis de la red asistencial La Libertad, con una muestra reducida por lo que el número de pacientes con estado nutricional normal fue muy bajo en comparación con la cantidad de pacientes con desnutrición, lo que impide una comparación adecuada. Además, el grupo poblacional de estudio es asegurada, lo que no corresponde necesariamente a una muestra homogénea de toda la población con ERC en hemodiálisis (asegurada y no asegurada).

En conclusión, se evidencia que, en nuestro entorno, la desnutrición es un problema de salud altamente frecuente entre los pacientes con ERC grado V que reciben hemodiálisis, predominando la desnutrición moderada a severa. Este hallazgo es importante ya que la desnutrición es un predictor de morbimortalidad y diversas complicaciones. Además, está relacionado a un mal pronóstico, incluida una menor calidad de vida, anemia refractaria y tasas significativamente mayores de hospitalización y mortalidad.
